# Clinical results at 10 years of minimum follow-up with the ABG 2 hip arthroplasty, matched with ceramic-on-ceramic bearings

**DOI:** 10.1051/sicotj/2022032

**Published:** 2022-08-15

**Authors:** Remy Coulomb, Jad Mansour, Jérome Essig, Gérard Asencio, Pascal Kouyoumdjian

**Affiliations:** 1 University Hospital of Nîmes Rue du Pr. Robert Debré 30029 Nimes cedex 9 France; 2 Clinique Médipole-Garonne 45, rue Gironis 31036 Toulouse cedex 1 France; 3 Laboratory of Mechanics and Civil Engineering (LMGC), CNRS-UM1 860 Rue de St-Priest 34090 Montpellier France; 4 Université Montpellier 1 2 Rue de l’École de Médecine 34090 Montpellier France

**Keywords:** THR, Hip arthroplasty, Uncemented, ABG II, ABG 2, Hydroxyapatite coating, HA

## Abstract

*Introduction*: The current study aimed as a primary goal is to assess the results of a ceramic-on-ceramic (CoC) bearing hip system matched with ABG (Anatomic Benoist Girard) 2 components in terms of survivorship. Secondary objectives addressed specifically ceramic-related complications as well as specific patterns at the bone-implant interface. *Material and methods*: This is a retrospective bicentric continuous series involving 147 patients (95 males vs. 52 females) who underwent ABG 2 arthroplasties with CoC bearings. One hundred and twenty-five hips were closely followed-up at a mean period of 11.3 years. *Results and discussion*: With a mean follow-up of 11.3 years, nine cases (5.7%) underwent revision surgery, four caused by acetabular aseptic loosening, three by deep infections, one ceramic head fracture, and one femoro-acetabular impingement. The global survivorship was 92.2% at 12.7 years. The Harris Hip Score (HHS) mean scores increased post-operatively from 50.1 up to 96.1 points (*p* < 0.001). All stems featured patterns of radiological osseous integration onto the hydroxyapatite (HA)-coated zones. No radiological wear or osteolysis of ceramic bearings was demonstrated however, five patients reported hip squeaking using this bearing. This study demonstrated excellent results at mid-term follow-up in patients younger than 70 years of age using cementless ABG 2 components coupled with CoC bearings with no increase in complication rate.

## Introduction

Primary total hip arthroplasty (THA) surgery is a successful and cost-effective surgery used to treat end-stage hip osteoarthritis and improve the patient’s daily function and quality of life [1]. However, this surgery can be associated with early and late complications such as dislocation, peri-prosthetic fractures, infection, or loosening [2, 3].

Various modern-bearing couples have been associated with adverse outcomes and decreased implant longevity. Many tribologic components have been implemented to decrease wear rates and challenge the complication of early revision surgery [4].

Using ceramic-on-ceramic (CoC) bearings as a standard procedure possibly decreases wear rates and enhances patients’ quality of life and prosthetic lifespan. The tribologic specifications and enhancements in third-generation ceramic components have allowed CoC to be considered a gold standard in total hip replacement surgery. This is possible because of its increased hardness and scratch resistance, thus reducing the volumetric wear debris compared to other bearing types.

The ABG 1 hip arthroplasty (Anatomic Benoist Girard, Stryker Orthopaedics^®^, Mahwah, NJ, US) has been widely used in Europe since 1985. Reported clinical results have been very good in the long run [5, 6]. Excellent radiological results have been reported with regards to the stem itself; however, a significant rate of osteolytic patterns behind the cup has been experienced as related to the polyethylene wear [7, 8]. Consistent modifications have been carried out while designing the new ABG 2 Hip System, aiming to lower the wear of bearings and enhance the quality of results. The ABG 2 hip stem, matched with a second-generation polyethylene on the acetabular side, as ceramic-on-polyethylene (CoP) bearings, led to acetabular osteolysis behind the cup getting avoided, and the femoral bone stock saved at best [9].

The current study aimed as a primary goal is to assess the results of a CoC-bearing hip system matched with ABG 2 components in terms of survivorship. Secondary objectives addressed specifically ceramic-related complications as well as specific patterns at the bone-implant interface.

## Material and methods

### Patients

This current study was a continuous retrospective series. All procedures were performed by two senior surgeons. Inclusion criteria included patients younger than 70 years with a minimal follow-up of 10 years and the exclusive use of a hydroxyapatite (HA)-coated ABG 2 hip system matched with CoC bearings.

**Figure 1:**
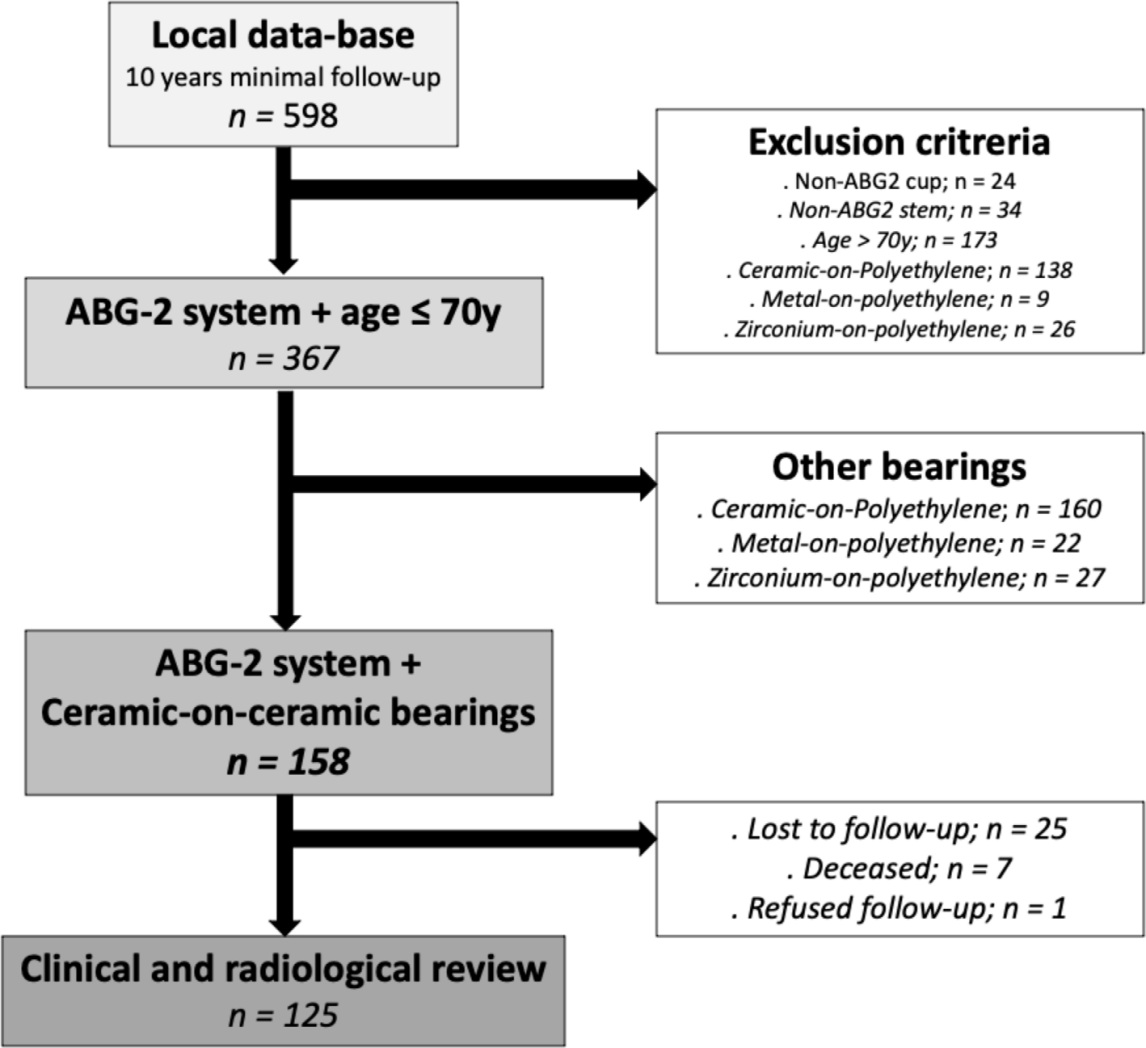
Participant flowchart.

In total, 158 hip replacements (147 patients) fulfilled the inclusion criteria: Ninety-five males versus 52 females in a follow-up period of 10 years. The average age was 53.3 years ± 9.8 years (22–70). The average body mass index (BMI) was 27.6 ± 4.8 (18.9–41.3). Twenty-two cases were bilateral. Etiologies are listed in Table 1. Thirty-one patients were excluded from the study, seven were deceased for non-surgical reasons, 23 were lost to follow-up (15.7%, 25 hips), and one patient refused follow-up. At the final follow-up, 116 patients (125 hips) were eligible for the study. The patient flow chart is shown in Figure 1.

**Table 1:** Etiology of surgery.

Etiology	Number of hips (*n*)	Percentages (%)
Primary hip arthritis	115	73
Osteonecrosis	30	19
Rheumatoid arthritis	3	2
Post traumatic arthritis	4	2
Congenital hip dysplasia	6	4

### Implants

All patients underwent a THA using a hydroxyapatite-coated ABG 2 total hip prosthesis (THA) (Stryker Orthopaedics^®^, Mahwah, NJ, USA). The fully HA-coated ABG 2 acetabular hemispherical shell was made of titanium alloy (TMZF). The alumina ceramic liner (Biolox^®^ forte, Ceramtec, Germany) had a 28 millimeters inner diameter in all cases. The stem had an anteversion of 7° and an anteversion of 5°. The morse taper (V40^TM^, Stryker Orthopaedics^®^, Mahwah, NJ, USA) was matched with a 28 mm alumina ceramic head and liner in all cases (Biolox^®^ Forte, Ceramtec, Germany).

### Surgical procedures

All surgeries were performed through a postero-lateral approach. The final acetabular implant was fitted size to size using a “press fit” technique. The cup positioning matched at best the native anteversion of the osseous cavity, and the inclination was given in the range of 40–45°.

On the femoral side, optimal preparation was obtained using rasps, of which the size was increased step by step. The need for previous diaphyseal reaming to calibrate the bed of the stem was anecdotical. The landmark used for assessing the right level of penetration of the rasp as given through the preoperative planification was mostly in accordance with the positioning of the stem shoulder at the level of the subtrochanteric fossa. The stem corresponding to the used rasp at final preparation got fitted under a press-fit mode. At the final step of surgery, the capsule was systematically sutured and external rotators reinserted. Additional steps were added in seven cases (4.4%), five patients underwent acetabular roof augmentation, one underwent acetabular grafting, and one in which a femoral cerclage was added prophylactically. Full weight-bearing was allowed in all cases on day 1 post-operatively with no ambulatory aid.

### Methods of evaluation

All clinical and radiological data, as well as the statistical analysis and the review of X-rays, were treated with the help of the OrthoWave software database (ARIA sas, Houdain 62150, France) according to the so-called “prospective-retrospective” protocol for data retrieval from a health data warehouse and covered by the official regulation on such type of research. All patients have given their consent before participating in the computerized study in each center. This study was approved by an institutional review board 11/05-03.

Clinical and radiological assessments were systematically performed preoperatively, at 3 months and 12 months following surgery, and yearly thereafter. This current study has been carried out by a unique clinical research assistant, who was independent of the surgeries at the latest follow-up, clinical and radiological controls were performed by a unique reviewer.

### Clinical and radiological assessments

Both Merle-D’Aubigné-Postel [10] and Harris hip score [11] was computed preoperatively and post-operatively. A satisfaction score was assessed as well at each follow-up visit. Radiological examination using pelvic and hip X-rays was performed, and acetabular and femoral parameters were computed by an independent observer. Stress shielding and radiolucent lines at the bone-implant interface were in the acetabular zones using the De Lee-Charnley classification [12] and on the femoral zones according to the Gruen classification [13]. Linear wear was computed using the Livermore method [14]. Heterotopic ossifications around the joint were assessed using the Brooker classification [15].

### Statistical analysis

Statistical analyses were performed using non-parametric tests. Cumulative survival rates were measured using the Kaplan-Meier method [16], with a 95% confidence interval. Endpoints were assigned as revision for any cause in any of the two components of the prosthesis. The significance level was appointed at 5% for all performed tests.

## Results

### Survivorship

**Figure 2:**
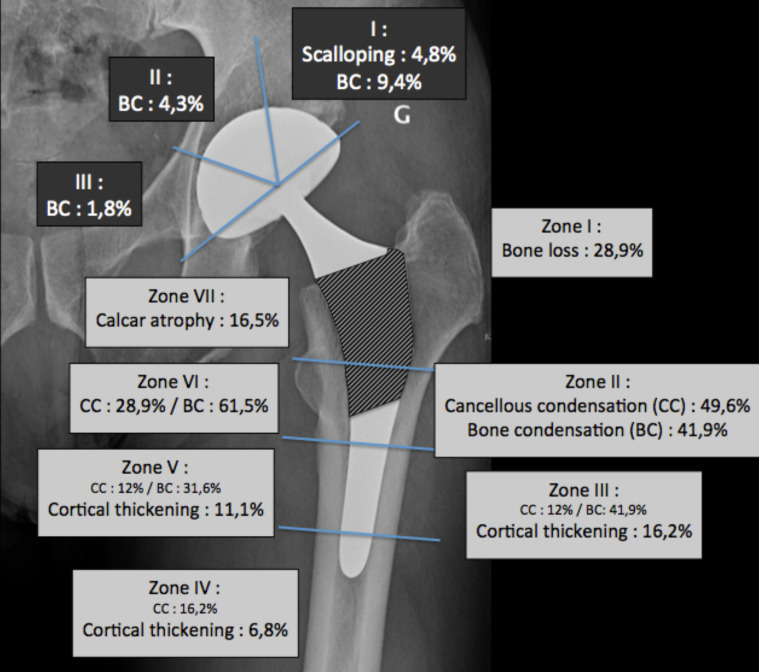
Survivorship of implants with revision surgery for any reason using Kaplan-Meier.

**Figure 3:**
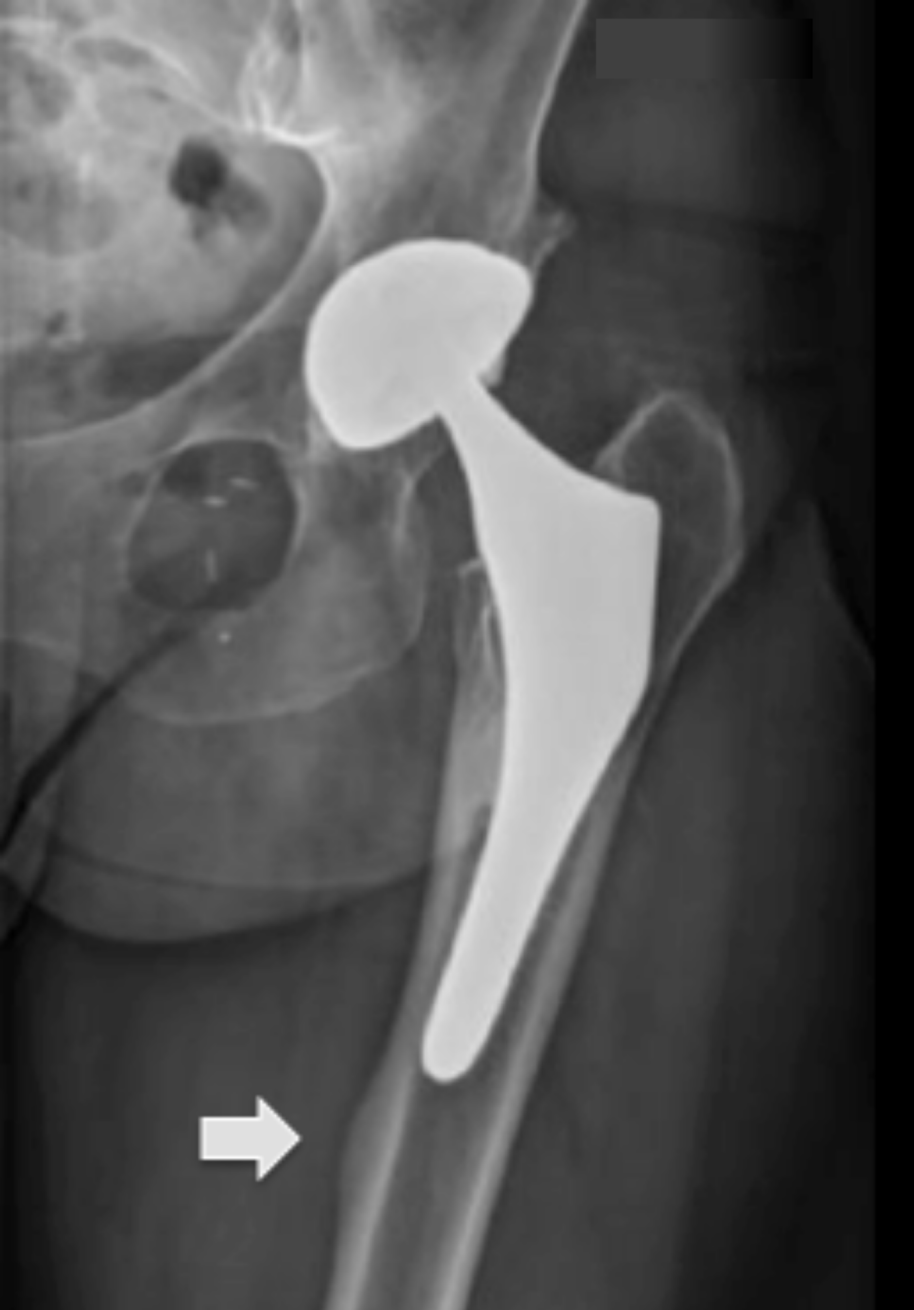
Survivorship aseptic loosening of aseptic loosening using Kaplan-Meier.

**Figure 4:**
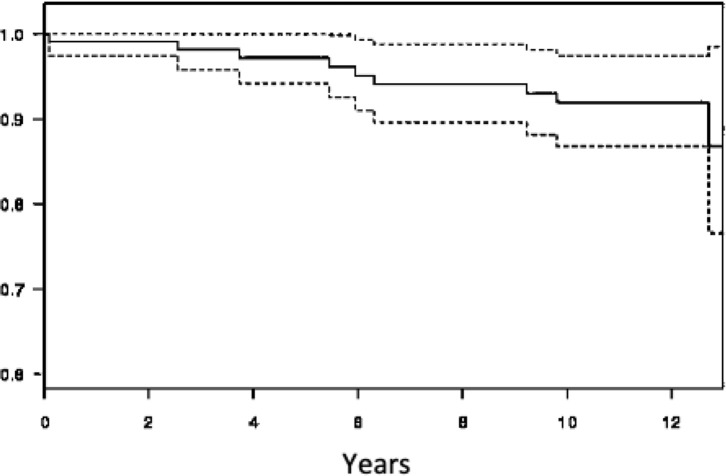
Survivorship of femoral stem aseptic loosening using Kaplan-Meier.

Taking revision surgery as the endpoint, the global survival rate of both the acetabular and femoral components was 92.2% (87.1–97.6) at 12.7-year (Figure 2). If aseptic loosening is considered the endpoint, the cumulative survival rate was 95.7% (91.6–100.0) for the cup and 100% for the stem, respectively (Figures 3 and 4).

### Complications (Table 2)

Of the 158 hips (147 patients), 14 complications (8.9%) were considered as none implant-related:

- Four cases of deep venous thrombosis (2.5%), two of them complicated by pulmonary embolism (1.3%).
- Five (3.2%) hip dislocations in four patients. They underwent closed reduction; one patient experienced two other recurrent dislocations. None of the cases required revision surgery.
- Three periprosthetic infections (i.e., two acute and one chronic: 1.9%). Two were infected 4 weeks post-operatively, treated by a one-stage revision surgery and intravenous antibiotics. The third case presented with an infected hematoma post-operatively, treated by synovectomy, antibiotics, and lavage followed by a periprosthetic femoral Vancouver C fracture, 8 years post-operatively fixed by open reduction and internal fixation using plate and screws.

**Table 2:** Complications and adverse events.

	*n*	%
Not implant-related		
Venous disease		
Deep veinous thrombosis	4	2.5
Pulmonary embolism	2	1.3
Dislocation	5	3.2
Deep infection	3	1.9
Fracture	1	0.6
Femoral acetabular conflict	1	0.6
Implant-related		
Aseptic loosening		
Cup	4	2.5
Stem	0	0
Implant breakage	1	0.6
Hip squeaking	5	3.2

The implant-related adverse events were:

- An atraumatic fracture of the ceramic head (offset: −2.7 mm), this complication was found at 13 years follow-up following squeaking sounds leading to revision.
- Four aseptic loosening of the acetabular cup (2.5%): Three (1.9%) demonstrated repetitive groin pain following a fall. A progressive radiolucent line was observed around the acetabular cup led to an isolated acetabular component revision. The fourth acetabular loosening was due to a local conflict between the cup screw head and the ceramic liner. At revision, a significant metallosis was recorded, and only the acetabular revision was performed.
- Five patients reported hip squeaking without any clinical implications, and no acetabular cup malpositioning was noted. There was no fracture of the ceramic bearings on the control Computed Tomography (CT) scan. None of them underwent revision surgery.

### Clinical and radiological assessments

Clinical and radiological results were assessed on all 125 hip implants (116 patients) at the last follow-up.

The Harris Hip Score (HHS) was statistically increased from 50.09 (15–81, *SD* = 13.6) preoperatively to 96.16 points (61–100, *SD*: 6.9) at the last follow-up (*p* < 0.001). The MDA score also increased from 8.79 (1–15, *SD* = 2.4) preoperatively to 17.42 (13–18, *SD =* 1.05) (*p* < 0.001). At review, patients were classified as very satisfied (86.4%), satisfied (12.8%), and unchanged (0.8%).

Radiologically, the mean acetabular cup inclination angle was 45.7° (30–59, *SD* = 6.0), while the mean cup anteversion was 24.9° (7–47, *SD =* 7.7). Radiolucent lines were assessed around the acetabular cup and femoral stem. Six (4.8%) were reported in zone 1 of the acetabular cup according to the De Lee-Charnley classification.

**Figure 5:**
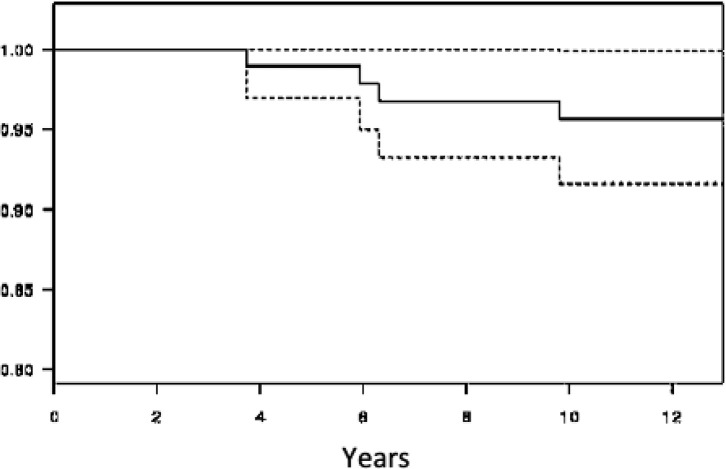
Radiological patterns at bone-implant interface. The cross-hatched area of the stem corresponds to the hydroxyapatite-coated area.

**Figure 6:**
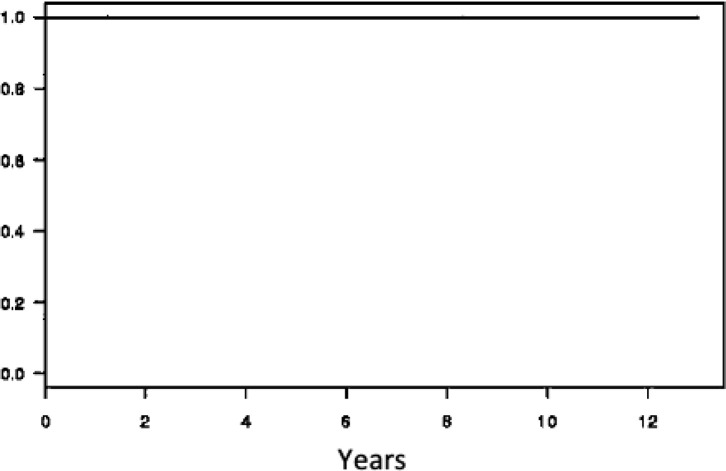
Cortical hypertrophy observed in zone 4. The white arrow shows stress shielding on the tip of the stem.

No femoral stem loosening was recorded. According to Gruen, patterns of radiological intimate bone apposition (Figure 5) were confirmed for all stems at HA-coated portions onto zones 2 and 6. At the proximal portion of the metaphyseal bone ongrowth, mild radiolucent lines were observed in 28.9% of hips in zone 1, as well as moderate calcar atrophy in zone 7 in 16.5% of the population. Mild cortical hypertrophy was recorded in 16.2% of hips on the distal uncoated portion of the femoral stems (Figure 6). No radiological loosening at review was observed according to Engh and Massin criteria. The linear wear computed was 19 μ/year for the acetabular cups; being said that in 48.7% of hips (*n* = 57), no wear was perceptible. Heterotopic ossifications were found in 13 hips (10.4%) according to Brooker classification, i.e., 10 hips in grade 1, two in grade 2, and one in grade 3.

## Discussion

This study highlights the excellent results at a minimum 10-year follow-up of the ABG 2 system associated with CoC bearing in patients less than 70 years old.

Wear-related failure is the most common reason for revision in many published series and joint arthroplasty registries and is significant for young patients. The overall revision may be caused by several etiologies, the most common cause was hip instability and liner fracture [17]. With the use of the ABG 1 system, the results reported by Tonino and Rahmy [5] and Blacha [6] demonstrated excellent fixation of the stem; this was related to femoral bone ongrowth. However, peri-acetabular osteolytic lesions have been highlighted with this system related to polyethylene wear [7, 8]. For these reasons, implementing the ABG 2 prosthetic components with ceramic heads and second-generation polyethylene liners bearings decreased these lytic reactions [9, 18, 19]. Addressing novel ceramic on ceramic bearings for younger patients propels a longer lifespan for this bearing couple.

Ferreira et al. [20] reported a global survival rate of 95.1% at 13 years with a third-generation CoC bearing. Moreover, Catanach et al. [21] reported 93.7% survivorship at a 6.58-year follow-up using the ABG II system. The main cause of failure of this system was associated with periprosthetic fracture and recorded 76% of complication cases related to those fractures. Thien et al. [22] also found a significant increase in the risk of fractures using the ABG 2 compared to uncemented femoral stems. In our series, with the cementless ABG 2 system, the survivorship rate of the ABG 2 system is comparable to the literature, reaching a rate of 92.2% at a 12-year follow-up. Our series reported no significant increase in periprosthetic fracture; only one fracture was observed. Nourissat et al. also reported one periprosthetic fracture [19] and Herrera et al. reported no cases of fracture [9].

Ceramic on ceramic bearings increased in use because of an increase in hardness and scratch resistance; they were found to decrease volumetric wear debris compared to the other bearing types. However, their use is still controversial because of their increased cost, risk of squeaking, high aseptic cup loosening rate [23], and prosthetic head fracture [4]. In our current study, the CoC couple wear rate was observed in 51.3% of cases and exhibited linear mean wear of 19 μ/year. However, there was a high intra- and inter-observers variability in the measurements mainly related to difficulty visualizing the joint line due to the opacity of implants and the tiny values of wear to be assessed. Hamadouche et al. [24] reported no radiological wear with CoC bearings at 18.5 years of minimum follow-up. In addition, Affatato et al. [25] reported an increased wear rate using in vitro testing with the use of Biolox^®^ Forte CoC bearing at a mean period of 13.2 years.

In addition, our series reported one case of ceramic head fracture (0.8%) with no ceramic liner fracture. This complication was observed 13-year post-operatively with no traumatic event. This patient underwent both component revision surgery, and a CoC bearing was placed. This complication rate is consistent with the ceramic head fractures reported by the manufacturer, i.e., 1.9 per 10,000 implants. Additionally, our series reported five cases (4.3%) of hip squeaking without any implant defect or clinical implications, and none of these patients underwent revision surgery. These implant-specific complication rates are close to those found by Tozun et al. [26].

Nourissat et al. [19] reported evidence of a so-called “radiological silence” around the ABG 2 acetabular cup at an 8-year follow-up. Those results were consistent with our study, which reported no radiological changes in 83% of cases. Taking aseptic loosening as the result, the survival rate of the acetabular cup in our series was reported as 95.7% at a follow-up of 8 years with 4 cases of aseptic loosening. We do not find in this study the catastrophic aseptic cup revision rates reported by Van Loon et al. [23]. Kim et al. [27] reported no significant difference between Ceramic on Ceramic and Ceramic on cross-linked Polyethylene bearing in terms of survivorship or aseptic loosening rates at 12 years of follow-up.

Our study reported complete osteointegration of our cases using the ABG 2 stem. This was consistent with the work published by Herrera et al. [9] and Van der Wal et al. [28]. These authors reported an increase in bone mineral density in zone 6 at a 2-year follow-up using a dual-energy X-ray absorptiometry (DEXA) scan. Moreover, based upon DEXA analyses, Kim et al. [29] did not find any influence of the bearing types (CoC vs. CoP) on the bone density of the proximal femoral shaft. In addition to finite element and DEXA studies, as reported by Gracia et al. [30], modifications featured to the ABG 2 stem allowed for decreasing the proximal stress-shielding as compared to ABG 1.

Aro et al. [31] reported in their study a minor migration of the ABG 2 stem with mean values of 0.9 mm at 3 months. Our study reported a radiological stress shielding at the proximal portion of the femoral shaft in one-third of cases and a decrease in cortical bone density at the distal portion of the femoral stem in 15.2%. This phenomenon has also been described by Herrera et al. [9], which considered bone porosity in 29.5% of their cases. Finally, our excellent stem survivorship is consistent with a study performed by Hailer et al. [32] that reported a 99% survival rate at a 10-year follow-up using 8872 ABG 2 stems using the Nordic Arthroplasty Register Association (NARA) database.

This study has limitations. One limitation is its retrospective analysis. However, the series was continuous with prospective data collection and a low rate of loss to follow-up at 10 years (25/15.8%). This retrospective analysis only slightly biases the main objective of the study, the analysis of system survival. Another limitation is the radiographic analysis which is less accurate than a CT analysis but reflects our current practice. To our knowledge, after Aro et al. [31] and their RSA study at 2 years, this study is the first to specifically assess and report outcomes of exclusive CoC bearings with the ABG 2 system at ten years of minimal follow-up.

In conclusion, this study demonstrated excellent results at mid-term follow-up in patients younger than 70 years of age using cementless ABG 2 components coupled with CoC bearings with no increase in complication rate. Additional long-term randomized studies will be necessary to confirm this increase in survival rates of such implants and decrease in complication rates.

## Conflicts of interest

Financial interests: Author Remy Coulomb, J Mansour, J Essig, and G Asencio declare they have no financial interests. Author Pascal Kouyoumdjian has received consultant honoraria from Company Stryker and Company Lepine. Pr. Kouyoumdjian has received a speaker honorarium from Company Stryker and Company Lepine.

## Funding

This research did not receive any specific grant from funding agencies in the public, commercial, or not-for-profit sectors.

## Ethical approval

This study was approved by an institutional review board 11/05-03.

## Informed consent

All patients have given their consent before participating in the computerized study in each center.

## Authors contribution

***R. Coulomb*** and ***G. Asencio***: Conceived and planned the experiments. ***G. Asencio***, ***J. Essig*** and ***P. Kouyoumdjian***: Performed surgery. ***R. Coulomb***, ***J. Essig***, ***G. Asencio***, and ***P. Kouyoumdjian***: Contributed to the interpretation of the results. ***R. Coulomb*** and ***J. Mansour***: Wrote the manuscript. All authors provided critical feedback and helped shape the research, analysis and final version of the manuscript.
